# Remotely Supervised Cranial Electrical Stimulation and Clinical Pain for Older Adults With Knee Osteoarthritis

**DOI:** 10.1093/geroni/igab046.632

**Published:** 2021-12-17

**Authors:** Hyochol Ahn, Hongyu Miao, Yumna Ali

**Affiliations:** 1 University of Texas Health Science Center School of Nursing, Houston, Texas, United States; 2 The University of Texas Health Science Center at Houston, Houston, Texas, United States

## Abstract

Knee osteoarthritis (KOA) is one of the most prominent causes of chronic pain, functional impairment, and disability in older adults. The current standards of care for KOA are aimed toward reducing pain and are largely comprised of analgesic medications, but existing pharmacologic approaches often produce significant adverse effects. Moreover, recent evidence suggests that KOA pain is characterized by alterations in pain-related brain mechanisms. Cranial electrical stimulation (CES), which delivers a low-amplitude alternating electric current to the brain, can facilitate the reversal of maladaptive brain function. Portable CES devices can be used at home with real-time monitoring through a secure videoconferencing platform to facilitate high adherence. Thus, the purpose of this pilot clinical study was to examine the preliminary efficacy of remotely supervised CES on clinical pain severity in older adults with KOA. Thirty participants with KOA were randomly assigned to receive 10 daily sessions of remotely supervised CES with 0.1 mA at a frequency of 0.5 Hz for 60 minutes (n=15) or sham CES (n=15). We measured clinical pain severity using the numeric rating scale (NRS; range, 0 – 100). Participants (67% female) had a mean age of 59 years. Active CES significantly reduced scores on the NRS (Cohen’s d = 1.43, P < 0.01). Participants tolerated CES well without any adverse events. Our findings demonstrate the promising clinical efficacy of remotely supervised CES for older adults with KOA. Future studies with larger-scale randomized controlled trials with follow-up assessments are needed to validate and extend our findings.

